# Supplementary material for: Altered microRNA Transcriptome in Cultured Human Liver Cells upon Infection with Ebola Virus
Source: Int J Mol Sci. 2021 Apr 6;22(7):3792. doi: 10.3390/ijms22073792 (PMC8038836; doi:10.3390/ijms22073792)
Supplement: Supplementary file 1 [file ijms-22-03792-s001.zip › Supplementary_File/C_ GO_Analysis_Results/16-30nt_go_Makona-96h-Huh7_vs_Control-96h-Huh7_up.mature_mirna_targets/CC_FoldEnrichment.pdf]

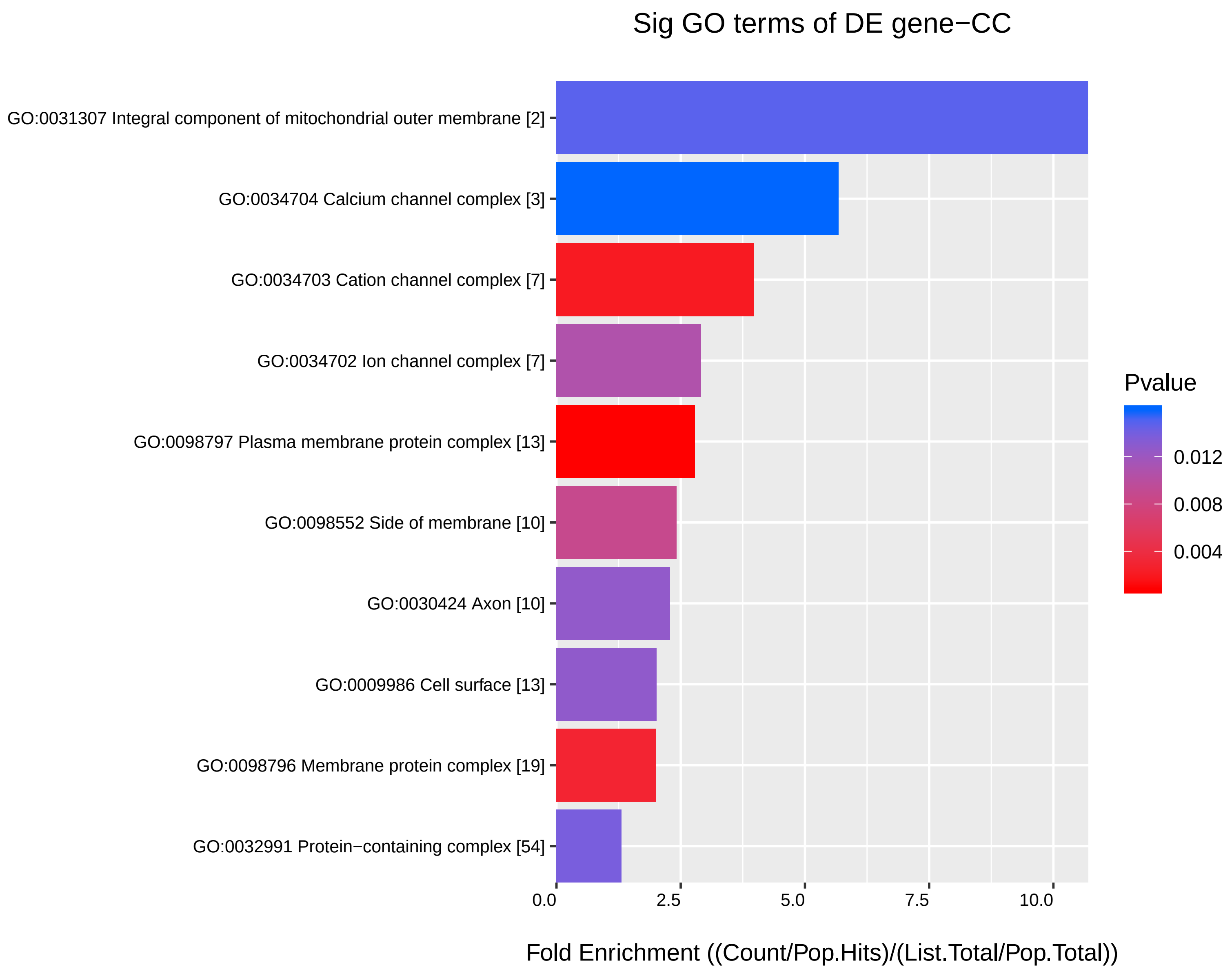

Sig GO terms of DE gene−CC
GO:0031307 Integral component of mitochondrial outer membrane [2]
GO:0034704 Calcium channel complex [3]
GO:0034703 Cation channel complex [7]
GO:0034702 Ion channel complex [7]
GO:0098797 Plasma membrane protein complex [13]
GO:0098552 Side of membrane [10]
GO:0030424 Axon [10]
GO:0009986 Cell surface [13]
GO:0098796 Membrane protein complex [19]
GO:0032991 Protein−containing complex [54]
0.0
2.5
5.0
7.5
10.0
Fold Enrichment ((Count/Pop.Hits)/(List.Total/Pop.Total))
Pvalue
0.012
0.008
0.004
